# Laparoscopic Cholecystectomy for Gallbladder Polyps: Is It Overtreatment?

**DOI:** 10.7759/cureus.68843

**Published:** 2024-09-06

**Authors:** Yalçın Burak Kara, Yahya Ozel

**Affiliations:** 1 Department of General Surgery, Bahcesehir University School of Medicine, Istanbul, TUR; 2 Department of General Surgery, VM Medical Park Pendik Hospital, Istanbul, TUR; 3 General Surgery, Dogus University School of Medicine, Istanbul, TUR

**Keywords:** benign polyp, cholecystectomy, gallbladder cancer, gallbladder polyps, pseudopolyps

## Abstract

Background: The gallbladder polyp (GP) is an accepted risk factor of gallbladder cancer and an indication for laparoscopic cholecystectomy (LC). Generally, the pathologic result of GPs is benign, but it is difficult to distinguish a potential malignancy or a stone without pathological evaluation. This study compared the indication and pathologic result of cholecystectomy performed due to GP in our clinic.

Materials and methods: This study employed retrospective data analysis. Patients who underwent LC from August 2021 through August 2024 were included in the study. Demographic features, operation status, indications for surgery, hospital stay, concomitant surgery, pathologic outcomes, and complications were recorded from patients’ data. Polyp sizes and number of polyps were taken from ultrasonography (USG) data.

Results: A total of 533 patients were included in the study. The mean age was 44.31 ± 12.14, and 64.35% (n = 343) were of female gender. Twenty patients (3.75%) underwent surgery for GP. The mean polyp size was 7.47 mm (2-15); 65% of the patients (n = 13) had multiple polyps, and 35% (n = 7) had a single polyp. The mean hospital stay was 1.59 ± 0.88 days. The pathologic result of GP was pseudopolyp in 55% (n = 11) of cases and non-polyp in 45% (n = 9). One patient (0.18%) who underwent an operation for gallstone had a malignancy. The sensitivity of USG in detecting polyps was found to be 64.7%. The complication rate was 1.5% (n = 8).

Conclusion: The pathological result of many patients who undergo cholecystectomy due to GPs is pseudopolyp or adenoma. In our study, no carcinoma was observed in any patient who underwent surgery for polyps. Further studies are needed to determine the indication for surgery due to GP.

## Introduction

Gallbladder polyps (GPs) are immobile lesions that grow from the gallbladder wall into the lumen. They are usually diagnosed incidentally while investigating the etiology of abdominal pain in ultrasonography (USG) or via a cholecystectomy specimen. The prevalence of GP varies from 0.3% to 9.5% [1-3]. Gallbladder stones and GPs are the main surgical indications of laparoscopic cholecystectomy (LC).

GPs may be classified as pedunculated or sessile, single or multiple, pseudopolyps, or true polyps. Nearly 70% of polyps are pseudopolyps. Cholesterol polyps, hyperplastic polyps, inflammatory polyps, and focal adenomyomatosis can appear as pseudopolyps, which are benign. True GPs may be benign (adenoma) or malignant (carcinoma) [1,2,4,5], but only 0.6% to 3% of GPs have malignant potential. The fact that GPs are one risk factor for gallbladder cancer makes their diagnosis, treatment, and follow-up important. Notably, the five-year prognosis of gallbladder cancer is as low as 14% in stage IIIB and IV tumors [3,6].

Because of the gallbladder’s nature, it is very difficult to distinguish a gallbladder lesion with radiological tools. USG diagnosis of gallstone achieves 95% accuracy by posterior acoustic shadow, echogenic focus, and gravity-dependent motion. Polyps are diagnosed when the patient moves and the lesion in the gallbladder remains motionless. The main goal is to differentiate pseudopolyps from true polyps with malignant potential. A posterior acoustic shadow with hypoechogenicity or izo-echogenicity is more likely to be a malignant feature [7-9]. In addition, various studies show that a sessile appearance, detected vascularity at the base of polyps, izo-echoic or hypoechoic condition, or focal thickening of the gallbladder wall may indicate that single polyps are malignant features [1]. Guidelines issued in 2017 define pseudopolyps’ ultrasonographic features as hyperechoic, multiple, pedunculated, and less than 1 cm in size [3].

The European Society of Gastrointestinal and Abdominal Radiology (ESGAR) and International Society of Digestive Surgery-European Federation (EFISDS) consensus guidelines recommend cholecystectomy if a GP is larger than 10 mm or the GP is 6-9 mm and accompanied by a high-risk factor, including age over 50, primary sclerosing cholangitis (PSC), Indian ethnicity, and sessile or focal wall thickening of over 4 mm (or an increase in sessile or focal wall thickening of 2 mm or more in the follow-up period) [3,5]. A study with over 1,200 patients with GPs found 20% malignancy in the operated group, and cholecystectomy was mandatory if the GP was over 10 mm [10]. In clinical practice, however, we are faced with pathologic results with the appearance of pseudopolyps in the gallbladder, raising the question of the necessity of surgery. The present study compared the indications and pathologic results of cholecystectomies performed for GPs in our clinic.

## Materials and methods

This research took the form of a retrospective data analysis study that included patients who underwent LC in Medical Park Pendik Hospital, Istanbul, from August 2021 through August 2024. We collected patient demographic features including age, gender, type of operation (open or laparoscopic), indications for surgery (gallstone, polyps, or part of a different operation), condition of surgery (emergency or elective, primary or concomitant surgery), pathologic evaluation of gallbladder, presence of preoperative endoscopic retrograde cholangiopancreatography (ERCP), length of hospital stay, and complications. If GPs were an indication for LC, the number of polyps (single or multiple) and their diameter were recorded from the patients’ USG. Complications in LC were defined as conversion to open surgery, bleeding, intra-abdominal abscess, and portal venous thrombosis. Indications for LC were compared for compatibility with the pathological evaluation of the gallbladder. The sensitivity and specificity of USG were calculated.

Inclusion and exclusion criteria

Patients who underwent LC, underwent an operation for symptomatic gallstone or GPs, or underwent cholecystectomy with a concomitant surgery, such as laparoscopic sleeve gastrectomy (LSG), laparoscopic gastric bypass, laparoscopic Nissen fundoplication, or gastric band removal, were included. Patients whose data could not be accessed or who had gallbladder removal as part of another surgery (Whipple procedure, total gastrectomy, and right hemi-colectomy) were excluded.

From August 2021 through August 2024, 548 patients underwent LC. Seven patients’ preoperative data were not accessible, and they were excluded. Eight patients were excluded who underwent cholecystectomy for a different malignancy, such as that of the pancreas, stomach, or right colon. Ultimately, 533 patients were included in the study.

Laparoscopic cholecystectomy technique

All the operations were performed by the same two surgeons, and the four-keyhole technique was used on the left side of all patients. After insufflation of the abdomen, Callot dissection was performed first by hanging the gallbladder with the help of an auxiliary trocar. The cystic artery and cystic duct were prepared separately, and the critical view of safety was achieved. After the cystic artery and cystic duct were clipped and separated, the surgery was completed by liver bed resection of the gallbladder.

Primary outcome of the study

This study’s primary outcome was to compare the indication for LC and the pathologic result to avoid unnecessary cholecystectomy.

Statistical analysis

The mean, standard deviation, minimum-maximum, median, frequency, and ratio values were used to obtain descriptive statistics. The Mann-Whitney U test, independent samples t-test, chi-squared test, and Fischer’s test were used to analyze quantitative and qualitative independent data, and the Kolmogorov-Smirnov test was used for variable distribution. A confusion matrix, sensitivity, and specificity test were measured with the help of artificial intelligence (ChatGPT, OpenAI, San Francisco, California). The results were also validated independently.

## Results

The study’s 533 patients had a mean age of 44.31 ± 12.14 years, and 64.35% (n = 343) were female. Among the patients, 96.25% (n = 513) underwent operations for gallstones, and 3.75% (n = 20) had surgery for GPs. In 438 cases (82.18%), LC was the primary operation, while 17.82% (n = 95) of the patients underwent concomitant surgery, including LSG, gastric bypass, Nissen fundoplication, and gastric band removal. The mean hospital stay was 1.59 ± 0.88 days. The pathologic evaluations of the gallbladder revealed cholecystitis due to stones (95.7%, n = 425), pseudopolyps (3.7%, n = 17), atypia (0.9%, n = 5), and adenocarcinoma (0.19%, n = 1). The complication rate of LC was 1.5% (n = 8) (see Table 1).

**Table 1:** Patient demographics SD: standard deviation, Min-max: minimum-maximum, GP: gallbladder polyp, LSG: laparoscopic sleeve gastrectomy, ERCP: endoscopic retrograde cholangiopancreatography, GIS: gastro-intestinal system

Variables		Value
Total patient count n		533
Age (years), mean± SD		44.31±12.14
Gender, n (%)	Female	343 (64.35)
	Male	189 (35.46)
Previous ERCP, n (%)	-	508 (95.31)
	+	25 (4.69)
Length of hospital stay (day), mean± SD, min-max		1.59±0.88 (1-7)
Length of hospital stay, n (%)	1 day	339 (63.6)
	2 days	91 (17.07)
	3 days	92 (17.26)
	4 days	9 (1.69)
	5 days	1 (0.19)
	7 days	1 (0.19)
Operation status, n (%)	Emergency	44 (8.26)
	Elective	489 (91.74)
Indication, n (%)	Gallstone	513 (96.25)
	GP	20 (3.75)
Type of operation, n (%)	Primary	438 (82.18)
	Concomitant	95 (17.82)
	+LSG	88 (16.51)
	+Gastric bypass	4 (0.75)
	+Funduplication	2 (0.38)
	+Gastric band removal	1 (0.19)
Complication, n (%)	-	525 (98.5)
	+	8 (1.5)
Type of complication, n (%)	Intra-abdominal hematoma	2 (0.38)
	Intra-abdominal abscess	1 (0.19)
	Converted to open surgery	2 (0.38)
	GIS bleeding	1 (0.19)
	LSG leak	1 (0.19)
	Portal vein thrombosis	1 (0.19)
Pathology, n (%)	Chronic cholecystitis	425 (79.74)
	Acute cholecystitis	85 (15.95)
	Pseudopolyp	17 (3.19)
	Atypia	5 (0.94)
	Adenocarcinoma	1 (0.19)

Twenty patients underwent an operation for GPs (3.75%), with a mean age of 37.95 ± 5.28 years. Thirteen of those patients (65%) had multiple polyps, while seven (35%) had a single polyp. The mean polyp size was 7.47 ± 3.72 mm (2.0-15.0).Three patients had additional sludge in the gallbladder. All patients were asymptomatic and underwent elective surgery, and the mean hospital stay was 1.25 ± 0.72 days. Pathologic evaluation of polyps revealed that 10 patients (50%) had cholesterol polyps and one patient (5%) had a hyperplastic polyp (a pseudopolyp) (55%), while nine patients (45%) had chronic stone-related cholecystitis. Neither true polyps nor malignancy was seen (as shown in Table 2).

**Table 2:** Characteristics of patients with gallbladder polyps SD: standard deviation, Min-Max: minimum-maximum, LSG: laparoscopic sleeve gastrectomy, ERCP: endoscopic retrograde cholangiopancreatography

Variables		Value
Total patient, count n		20
Age (years), mean±SD		37.95±5.28
Gender, n (%)	Female	10 (50.0)
	Male	10 (50.0)
Previous ERCP, n (%)	-	20 (100.0)
	+	0 (0.0)
Length of hospital stay (day), mean±SD, min-max		1.25±0.72 (1-3)
Length of hospital stay (days), n (%)	1 day	17 (85.0)
	2 days	2 (10.0)
	4 days	1 (5.0)
Operation status n (%)	Emergency	0 (0.0)
	Elective	20 (100.0)
Polyp (mm), mean±SD, n (%)	Size	7.4±3.72
	Multiple	13 (65.0)
	Single	7 (35.0)
Operation type, n (%)	Primary	19 (95.0)
	Concomitant (LSG)	1 (5.0)
Complications, n (%)	−	19 (95.0)
	+	1 (5.0)
Pathology, n (%)	Pseudopolyp	11 (55.0)
	Chronic cholecystitis	9 (45.0)

**Figure 1:**
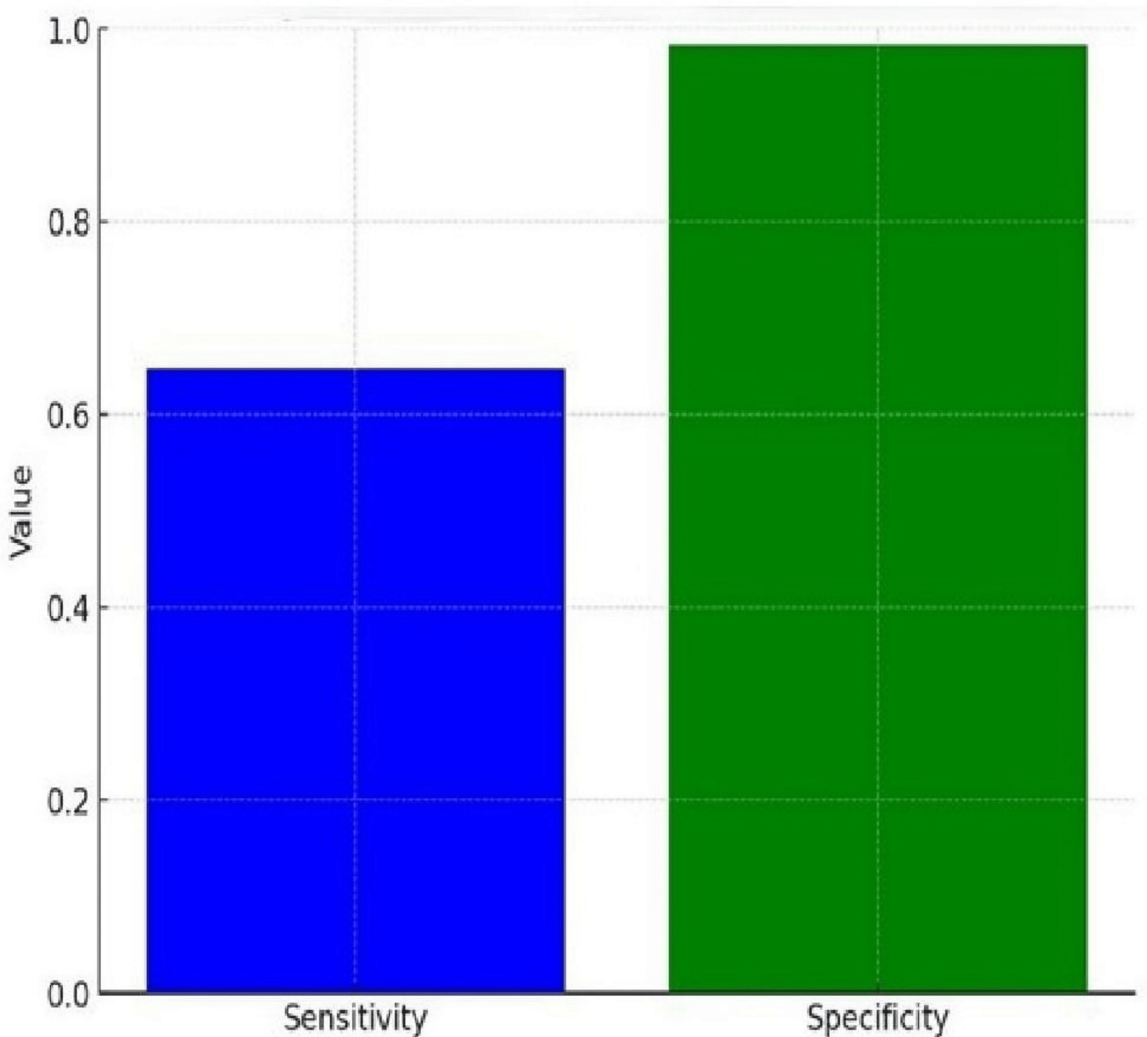
Sensitivity and specificity of ultrasonography (USG) for the detection of polyps

**Figure 2:**
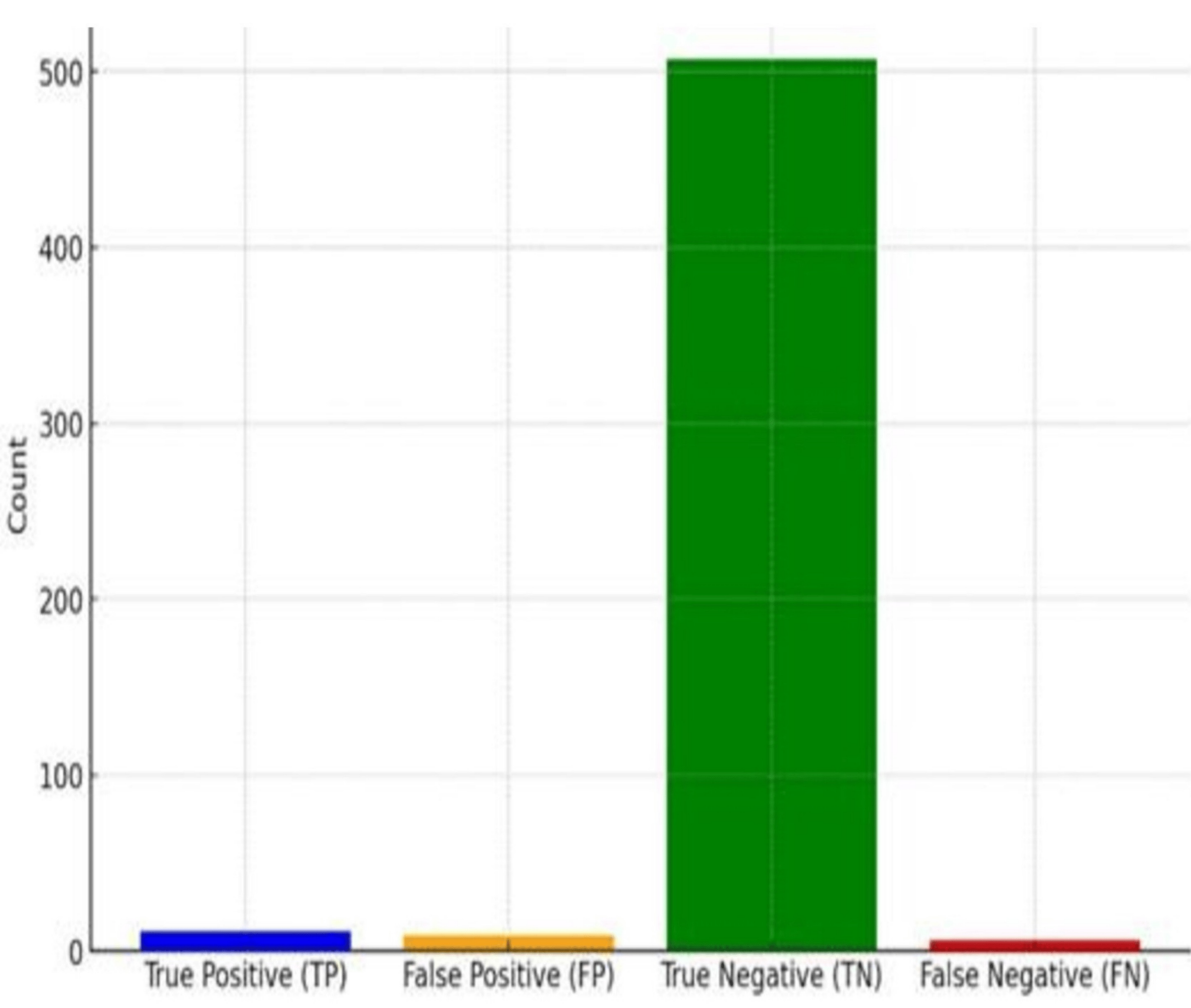
Counts of true-false positivity and negativity for polyp detection

**Figure 3:**
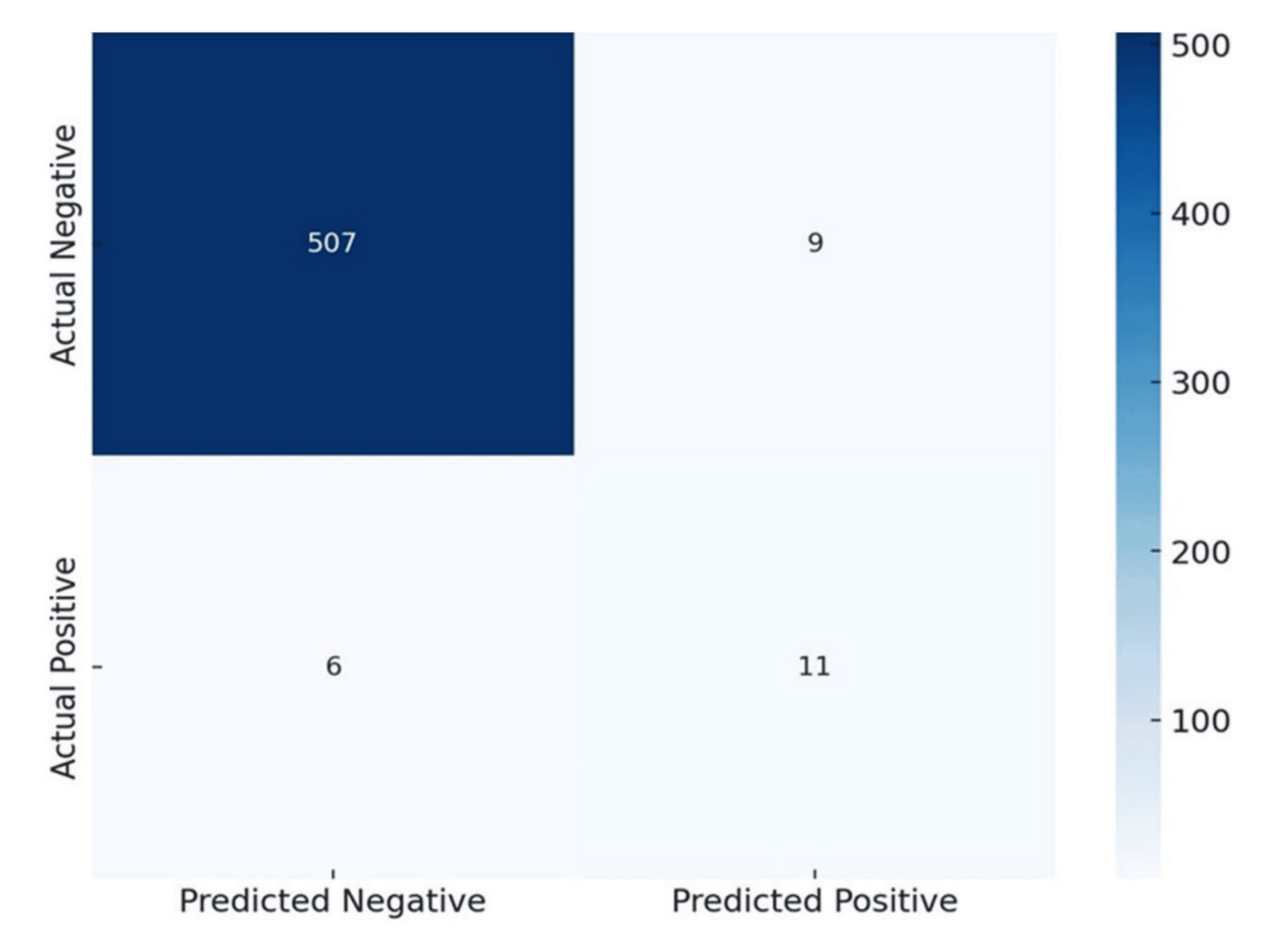
Prediction of polyps and stones by ultrasonography (USG)

Using USG, we calculated the following values for polyps: true positive (TP): 11/20 (55%), false positive (FP): 9/20 (45%), true negative (TN): 507/513 (98.8%), and false negative (FN): 6/513 (1.2%). The sensitivity and specificity for polyps were as follows: sensitivity: 0.65 (64.7%) and specificity: 0.98 (98.3%). These results indicate that USG has a moderate sensitivity for detecting polyps, correctly identifying polyps in 64.7% of cases. However, it has a very high specificity of 98.3%, meaning it accurately identifies non-polyp cases 98.3% of the time (shown in Figures 1, 2, 3).

**Figure 4:**
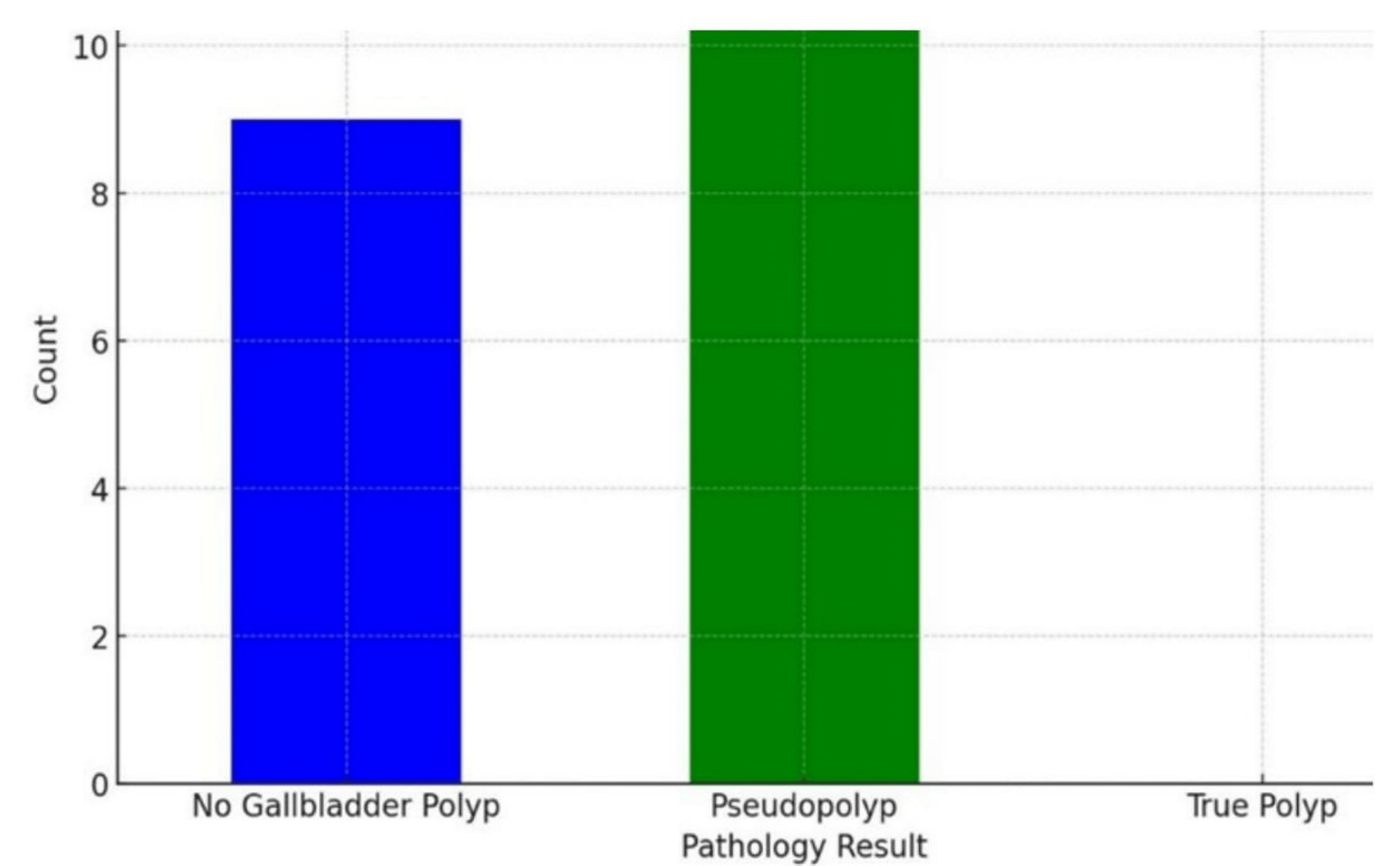
Pathologic results for patients with ultrasonography (USG)-detected polyps

Examination of the pathological results of the gallbladder for GP revealed 10 patients (50%) with cholesterol polyps and one patient (5%) with hyperplastic polyps; 55% (n = 11) of GP specimens were pseudopolyps. Nine patients’ (45%) pathological evaluation revealed chronic cholecystitis by gallstones, whereas their preoperative USG suggested GPs. There were no true polyps in the form of adenoma or carcinoma (see Figure 4).

Complications

The complication rate of all operations was 1.5% (n = 8). One (0.19%) intraluminal bleeding, one (0.19%) intra-abdominal hematoma (nearby sleeve line in CT scan), and one (0.19%) leakage were related to the concomitant surgeries of LSG and gastric bypass. The LC-related complication rate was 0.93% (n = 5). Two patients (0.38%) required conversion to open cholecystectomy, whose pathologic results were gallstones. One patient (0.19%) had postoperative bleeding, and a transfusion was performed, but re-laparoscopy was not needed. One patient (0.19%) had an abdominal abscess and required rehospitalization, antibiotherapy, and drainage under USG by our interventional radiology team. One (0.19%) case had a portal vein thrombosis. There was no bile duct injury and no mortality.

## Discussion

LC is one of the more commonly performed procedures in daily general surgical practice. Symptomatic gallstones and polyps are the main indications for cholecystectomy. GPs are incidentally seen in around 0.3-12.3% of the population, and polyps may appear as pseudopolyps (e.g., cholesterol polyps, hyperplastic polyps, inflammatory polyps, and adenomyomatosis) or true polyps (e.g., adenoma and carcinoma). GP is a risk factor for gallbladder cancer, which has a very poor prognosis. Hence, differential diagnosis of GP as pseudo or true polyps is crucial due to its malignant potential [4,10,11]. We tried to compare the indication for LC and the pathologic result to avoid unnecessary cholecystectomy.

The incidence of gallbladder malignancy varies by region. A review indicates that the malignancy incidence rate of GPs is 14.1% in Asia and 6.2% in Europe [12]. A systematic review of 20 studies with a total of 5,482 patients states that the incidence of malignancy in gallbladder specimens in operations for GP was 0.4% [4]. In our country, the incidence of incidental gallbladder cancer was 0.4% [13]. By contrast, the incidence of adenocarcinoma in GPs varied from 6.3% to 8.7% [12,14]. The malignancy rate of GPs may differ according to polyp size. Some studies indicate that malignancy was not observed if the polyp size was less than 5 mm, whereas the incidence of malignancy was 8-10% if the GP was over 10 mm [15,16]. A study with 438 patients suggested that the cut-off value for malignancy in GP was 21 mm and that for dysplasia was 11.8 mm [17]. We observed no malignancy in patients undergoing operation for GP in our study. While 533 cholecystectomies were performed in our study, only one patient, a 66-year-old male undergoing primary operation for gallstone, had a gallbladder adenocarcinoma.

The pathology results of most surgeries for GPs indicate benign polyps. A systematic review found that 69.6% of pathology results in gallbladder operations for GP indicated pseudopolyps, and nearly 16.4% were non-polyps [4]. Studies have found an incidence of non-polyp lesions varying from 12.9% to 17.5% [10,12]. A study of 115 patients who had undergone preoperative endoscopic ultrasound (EUS) for GP indicated a 78.3% rate of non-neoplastic polyps and a 1.7% rate of non-polyp lesions [15]. Gallstones and polyps may appear the same on an ultrasound. This confusion can be cleared up by using EUS. In our study, 45% of pathological outcomes of GP were non-polyps. For this reason, EUS can be used as a problem-solving modality to prevent unnecessary surgery.

The risk factors for gallbladder malignancy in ESGAR and EFISDS’ revised guidelines for GP include age over 60, history of PSC, Indian ethnicity, sessile polyps, and focal gallbladder wall thickening over 4 mm [3,18]. A systematic review found risk factors defined as size greater than 6 mm, growth in follow-up, single polyp, and Indian ethnic background [4]. In addition, the predictive factors for pseudopolyps may be defined as age under 48.5, polyp diameter less than 13.25 mm, and multiple polyps [12]. In our study, 65% of patients had multiple polyps, the mean diameter of polyps was 7.47±3.72 mm, and the mean age was 37.95±5.28 years. This could explain the high pseudopolyps percentage and absence of malignancy in patients undergoing operations for GP in our study.

Many radiologic methods are used to diagnose GPs. The sensitivity of USG for diagnosing GPs varies from 50% to 90.1% [16]. Some studies find that EUS is more successful than USG in diagnosing GPs. A study suggests that an EUS score based on morphologic findings, such as layer structure, echo pattern, margin, stalk, and number of polyps, yields statistically significant results in the detection of neoplastic polyps, especially those between 5 mm and 15 mm in size [18]. Furthermore, high-resolution ultrasonography (HRUS) provides the same efficacy as EUS in detecting neoplastic GPs [19]. Color Doppler flow EUS supports the differential diagnosis of GPs of 7-20 mm in size [14], and magnetic resonance imaging yields 76.9% sensitivity, 84% specificity, and 83% accuracy in identifying malignancy in GPs [15]. The ESGAR guideline suggests that an additional imaging modality, such as EUS, may be useful, but routine use of EUS is not recommended. In our study, all patients who underwent operations for GP were examined with conventional USG preoperatively by different radiologists. The pathologic result of 55% of patients who underwent LC due to GP was pseudopolyps, and 45% had gallstones. The sensitivity of USG was 64.7% in our study. These data show that additional images, such as EUS or other tests, are needed before operation, especially in GPs of 5-20 mm.

Many studies, systematic reviews, and guidelines (e.g., ESGAR) recommend surgery for GPs over 10 mm [1,3-5,10]. A study from the United Kingdom found that 75.9% of surgeons recommend surgery for single polyps over 10 mm, and 50.9% of surgeons recommend surgery for multiple polyps of less than 10 mm [20]. However, some surgeons use a nomogram for 10-15 mm GPs that considers the number of polyps, type of polyps, echogenicity, and size of the polyps, with a large/small diameter predicting the risk of malignancy [21].

The findings described above suggest that many unnecessary cholecystectomies have been performed for pseudopolyps or non-polyps above 10 mm. Rapidly performed ultrasonographic examinations may easily confuse gallstones and GP due to their use in a great number of patients. To avoid unnecessary cholecystectomy, a secondary check for risk factors or routine examination of GPs with additional imaging modalities before surgery, such as EUS or HRUS, may be added to the guidelines.

Limitations

This study has some limitations. The small sample size and single-center design limit the study, and the gallbladder cancer incidence was very low. The fact that patients applying for cholecystectomy had undergone USG by different radiologists in different centers reduced the effectiveness of USG. However, the fact that additional imaging methods, such as MRI or EUS, were not performed along with USG explains the high rate of pseudopolyps in the pathology results. We believe that more definitive results could be obtained by prospective design studies that include additional imaging methods.

## Conclusions

One of the reasons for cholecystectomies is polypoid lesions of the gallbladder. In our study, the pathologic result of cholecystectomy for GPs was 55% pseudopolyps and 45% non-polyps. True polyps or malignancy was not observed in the pathology of GPs.The malignancy rate of all cholecystectomies was 0.19%. Prospective randomized studies including surgical indication of gallbladder polypoid lesions and preoperative radiologic images are needed to avoid unnecessary cholecystectomy.
